# Second‐line treatment of hepatocellular carcinoma after sorafenib: Characterizing treatments used over the past 10 years and real‐world eligibility for cabozantinib, regorafenib, and ramucirumab

**DOI:** 10.1002/cam4.3116

**Published:** 2020-05-07

**Authors:** Andrea S. Fung, Vincent C. Tam, Daniel E. Meyers, Hao‐Wen Sim, Jennifer J. Knox, Valeriya Zaborska, Janine Davies, Yoo‐Joung Ko, Eugene Batuyong, Haider Samawi, Winson Y. Cheung, Richard Lee‐Ying

**Affiliations:** ^1^ Tom Baker Cancer Centre Calgary AB Canada; ^2^ Cumming School of Medicine University of Calgary Calgary AB Canada; ^3^ Princess Margaret Cancer Centre Toronto ON Canada; ^4^ NHMRC Clinical Trials Centre University of Sydney Camperdown NSW Australia; ^5^ BC Cancer Agency Vancouver Centre Vancouver BC Canada; ^6^ Faculty of Medicine University of British Columbia Vancouver BC Canada; ^7^ Sunnybrook Health Sciences Centre – Odette Cancer Centre Toronto ON Canada; ^8^ St. Michael's Hospital Toronto ON Canada

**Keywords:** cabozantinib, hepatocellular carcinoma, ramucirumab, regorafenib, trial eligibility

## Abstract

**Background:**

The CELESTIAL, RESORCE, and REACH‐2 trials showed survival benefit of cabozantinib, regorafenib, and ramucirumab, respectively, in hepatocellular carcinoma (HCC) patients treated with sorafenib who had good performance status (ECOG 0‐1) and liver function (Child‐Pugh‐A). This study characterizes subsequent treatments received by HCC patients after sorafenib, and determines the proportion of patients eligible for novel therapies if strict eligibility criteria (SEC) were utilized compared to more liberal modified eligibility criteria (MEC, including ECOG 2, Child‐Pugh‐B7).

**Methods:**

HCC patients who received sorafenib between 2008 and 2017 were included from the Canadian HCC CHORD Database. Patients were classified as eligible or ineligible based on available CELESTIAL, RESORCE, and REACH‐2 trial SEC or MEC. Median overall survival (mOS) was assessed using the Kaplan‐Meier method.

**Results:**

A total of 730 patients were identified; and 172 (23.6%) received subsequent treatment. Patients who received subsequent treatment had longer mOS than those who did not (12.1 vs 3.3 months; *P *< .001). Using SEC, only 13.1% of patients would be eligible for cabozantinib, regorafenib, or ramucirumab. Expanding eligibility to include patients who meet MEC increased the proportion of eligible patients to 31.7%. Higher ineligibility for regorafenib and ramucirumab was driven by trial‐specific criteria, including sorafenib intolerance (28%) for RESORCE and AFP <400 (58.9%) for REACH‐2.

**Conclusions:**

A small proportion of real‐world HCC patients would be eligible for cabozantinib, regorafenib, or ramucirumab if SEC in clinical trials were followed, while more than double would be eligible if MEC were applied. Patients who received subsequent treatment had improved mOS, regardless of whether they met SEC or MEC.

## Graphical abstract

Few real world HCC patients would be eligible for cabozantinib, regorafenib or ramucirumab if strict eligibility criteria utilized in clinical trials were used to select patients for treatment in clinical practice. We show that select patients with ECOG 2 performance status or Child‐Pugh B7 (CPB7) liver function might still benefit from treatment; therefore, modified eligibility criteria including ECOG 2 and CPB7 patients should be considered in clinical practice.

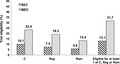

## 1 INTRODUCTION

Liver cancer is the second leading cause of cancer death and the Sixth most common cancer worldwide.^1^, ^2^ In Canada, there were 2500 diagnoses and 1200 deaths from liver cancer in 2017.^3^ Incidence rates of hepatocellular carcinoma (HCC) have been increasing in the United States, and despite decreasing death rates among more common cancers, the death rates associated with HCC have been increasing.^4^

Sorafenib has been the standard first‐line treatment of advanced hepatocellular carcinoma not amenable to locoregional therapy for over a decade.^5^, ^6^ Lenvatinib has recently shown noninferior survival compared to sorafenib in the first‐line setting.^7^ Numerous studies have failed to identify novel treatments with superior efficacy compared to sorafenib,^8^, ^9^, ^10^ although early results from the IMbrave 150 study suggest better survival with atezolizumab plus bevacizumab when compared to sorafenib.^11^ Until recently, there were limited options for treatment of HCC patients who had progressed on sorafenib. Recent randomized trials have shown survival benefit with cabozantinib, regorafenib, and ramucirumab when used after sorafenib, which has led to a change in the treatment landscape for patients with HCC.

The oral multikinase inhibitors cabozantinib and regorafenib, and the monoclonal antibody ramucirumab all target signaling through the VEGF/VEGFR pathway.^12^, ^13^, ^14^ Three phase III randomized trials studied these agents in HCC patients who had been previously treated with sorafenib, and showed a survival benefit when compared to placebo.

In the CELESTIAL trial, there was an improvement in median overall survival (mOS) (10.2 vs 8.0 months; HR 0.76, 95% CI 0.63‐0.92, *P *= .0049) and progression‐free survival (PFS) (5.2 vs 1.9 months; HR 0.44, 95% CI 0.36‐0.52, *P *< .0001) with cabozantinib when compared to placebo.^12^ Similarly, the RESORCE phase 3 randomized clinical trial compared regorafenib to placebo in HCC patients who had tolerated but progressed on sorafenib. mOS was 10.6 months for regorafenib compared to 7.8 months for placebo (HR 0.64, 95% CI 0.50‐0.79, *P *< .0001).^13^ Ramucirumab showed an improvement in mOS (8.5 months vs 7.3 months; HR 0.71, *P *= .0199) and PFS (2.8 months vs 1.6 months; HR 0.45, *P *< .0001) in the REACH‐2 trial when compared to placebo in HCC patients with an AFP of 400ng/mL or greater who had been previously treated with sorafenib in the first‐line setting.^14^

The strict eligibility criteria (SEC) utilized in these phase III randomized trials may limit generalizability of the results to patients being treated in the real‐world setting. The present retrospective analysis characterizes subsequent treatments received by HCC patients at multiple cancer centers across Canada over the past decade, and assesses their impact on survival in the real‐world setting. We also evaluate which patients in the real‐world would be eligible for novel treatments using SEC from the CELESTIAL, RESORCE, and REACH‐2 clinical trials, and determine the prognostic impact of using modified eligibility criteria (MEC) which would include patients with slightly worse performance status (ECOG 2) and/or limited liver dysfunction (Child‐Pugh‐B7).

## 2 METHODS

### 2.1 Study population

The HCC Cancer Health Outcomes Research Database (CHORD) consortium is a Canadian research initiative that seeks to pool real‐world data to study outcomes in HCC patients treated with systemic therapy. Standardized data elements are collected from each participating institution and then merged into a central repository. For this study, data were combined from cancer centers in three Canadian provinces: British Columbia, Alberta, and Ontario.

Patients with a confirmed diagnosis of HCC who were treated with sorafenib between January 2008 and June 2017 at all British Columbia Cancer Agency cancer centers in British Columbia, all cancer centers in Alberta, and 2 cancer centers in Toronto, Ontario (Princess Margaret Cancer Centre, Sunnybrook Odette Cancer Centre) were retrospectively identified. Clinical, pathologic, laboratory, treatment, and outcome data were collected from the CHORD consortium database. Ethics approval was obtained from each provincial cancer research ethics board or individual participating center as per local institutional guidelines prior to data collection for this study.

### 2.2 Subsequent treatments

The type of treatment received by patients after sorafenib was characterized. Localized treatments included stereotactic body radiation therapy (SBRT), transarterial chemoembolization (TACE), transarterial radioembolization (TARE), bland embolization, radiofrequency ablation (RFA), and surgical resection. Systemic therapies included chemotherapy and clinical trial drugs such as multikinase inhibitors, immunotherapy, or other trial drugs. Palliative radiation included radiation treatment to nonlocoregional sites.

### 2.3 Eligibility criteria

Patients who met the common clinical trial inclusion/exclusion criteria from the CELESTIAL, RESORCE, and REACH‐2 trials were identified from our database. The common trial inclusion criteria were ECOG performance status 0‐1, Barcelona Clinic Liver Cancer (BCLC) stage B or C, Child‐Pugh‐A, and absence of ascites.

Patient charts were also reviewed for trial‐specific inclusion criteria, which were each specific to only one of the clinical trials. RESORCE trial‐specific criteria included sorafenib tolerability (defined as ≥400 mg daily for at least 20 of the 28 days before discontinuation), as well as documented radiographic progression during sorafenib treatment. REACH‐2 trial‐specific criteria included an alpha‐fetoprotein (AFP) level of ≥400 ng/mL. Patients who met these trial‐specific inclusion criteria for each trial and the common inclusion/exclusion criteria listed above were defined as meeting SEC for that specific trial.

Patients included in the modified eligibility criteria (MEC) group included those in the SEC group, as well as patients with ECOG 2 performance status and/or limited liver dysfunction (Child‐Pugh‐B7). These patients, while not generally eligible for HCC clinical trials would be regarded by most oncologists as well enough for treatment in the real‐world setting.

### 2.4 Statistical analyses

The Kaplan‐Meier method was used to estimate overall survival (OS) based on subsequent treatment. For assessment of survival outcomes based on trial eligibility, patients were classified as eligible or ineligible based on available CELESTIAL, RESORCE, REACH‐2 clinical trial eligibility criteria (SEC), and also MEC.^12^, ^13^, ^14^ OS for these groups was compared using the log‐rank test. A multivariable Cox proportional hazards model was used to control for impact of eligibility criteria. All statistical analyses were performed using SPSS Version 23.0, Armonk, NY: IBM Corp.

## 3 RESULTS

### 3.1 Patient Characteristics

A total of 730 patients with HCC previously treated with sorafenib were identified. In this cohort, patients were treated with sorafenib for a median duration of 3.4 months, and received a mean dose of approximately 66.1%. Baseline patient characteristics are summarized in Table 1. Median age of the total population was 64 years. The majority of patients were male (n = 590; 80.8%), and were of non‐East Asian ethnicity (n = 471; 64.5%). Approximately half of the patients had a performance status of ECOG 0‐1 (42.9%), while 31.5% were ECOG 2 after stopping first‐line sorafenib. Only 45.9% of the patients had Child‐Pugh‐A liver function at the conclusion of their sorafenib treatment, while 20.1% were Child‐Pugh‐B7. The most common underlying etiologies of liver disease were viral hepatitis (HBV 32.2%, HCV 31.9%), followed by alcohol (22.1%) and nonalcoholic steatohepatitis (NASH; 6.8%).

**TABLE 1:** Baseline patient characteristics (at time of discontinuation of sorafenib)

Characteristics	Total population (n = 730)
Age (y)	
Median	64
Mean	64
Gender (%)	
Female	19.2
Male	80.8
Ethnicity (%)	
East Asian	35.5
Non‐East Asian	64.5
ECOG performance status (%)	
0	7.7
1	35.2
2	31.5
3	15.1
4	2.9
Unknown	7.7
Child‐Pugh (%)	
A	45.9
B (B7)	43.3 (20.1)
C	6.6
Unknown	4.2
Confirmed Histology (%)	
Yes	90.3
Etiology of liver disease (%)	
HBV	32.2
HCV	31.9
EtOH	22.1
NASH	6.8
Hemochromatosis	2.6
Alpha‐1 antitrypsin	0.4
Other	1.9
M Stage (%)	
M1	46.5
Ascites (%)	
Absent	65.1
Medically controlled	27.3
Poorly controlled	2.7
Unknown	4.9
Encephalopathy (%)	
Absent	92.7
Medically controlled	2.7
Poorly controlled	0.1
Unknown	4.4
AFP nadir—median	145
Bilirubin at last cycle of sorafenib (%)	
<34	96.2
>34	3.8
Sorafenib treatment	
Median treatment duration (months)	3.4
Mean sorafenib dose (%)	66.1
Reason for discontinuation of sorafenib (%)	
Toxicity	21.5
Patient choice	8.5
Disease progression	64.5
Death	3.7
Other	1.8

### 3.2 Subsequent Treatment after sorafenib

A total of 172 (23.6%) patients received subsequent treatment after sorafenib (see Table 2 for full details). Of the patients who received subsequent treatment, 32 (18.6%) had an ECOG 2 performance status or Child‐Pugh B7 liver function. Only 12.9% received systemic therapy and 7.3% received localized therapy. The majority of patients who received subsequent systemic therapy did so on a clinical trial (10.3%); 15 patients (2.1%) received cabozantinib, 10 patients (1.4%) received regorafenib, and none was treated with ramucirumab.

**TABLE 2:** Types of subsequent treatment received by HCC patients after sorafenib

Treatment type	Number of patients (%)^a^
Systemic	94 (12.9)
Chemotherapy	20 (2.7)
Clinical Trial	75 (10.3)
Axitinib	25 (3.4)
Cabozantinib	15 (2.1)
Regorafenib	10 (1.4)
Immunotherapy	9 (1.2)
Other trial drug	22 (3.0)
Localized	53 (7.3)
SBRT	21 (2.9)
TACE	17 (2.3)
RFA	12 (1.6)
Resection	6 (0.8)
TARE	4 (0.5)
Embolization	3 (0.4)
Palliative RT	37 (5.1)
None	552 (75.8)

^a^ Totals do not add up to 100% since some patients received multiple treatments. The total population (n = 730) was evaluated.

### 3.3 Eligibility for second‐line treatment based on strict eligibility criteria (SEC) versus modified eligibility criteria (MEC)

**FIGURE 1:**
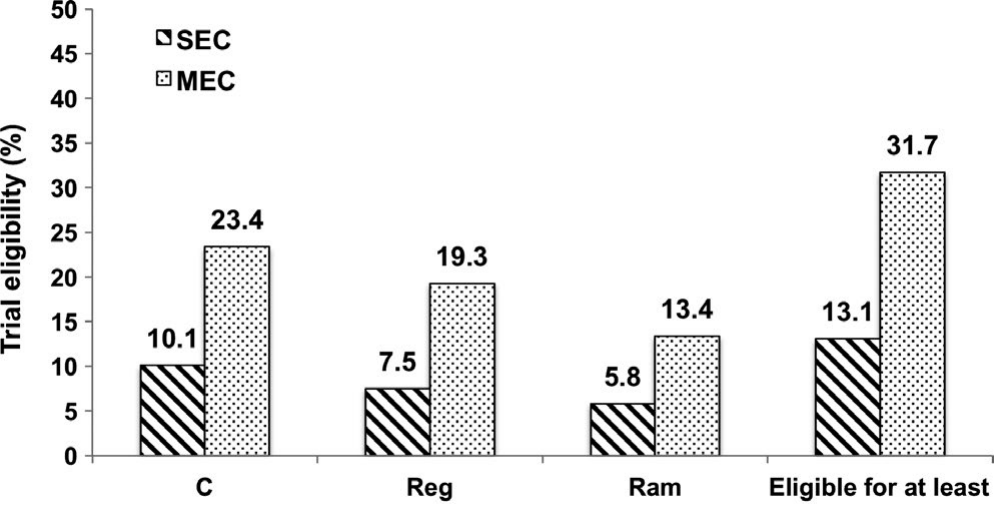
Eligibility for cabozantinib, regorafenib or ramucirumab based on strict eligibility criteria (SEC) and modified eligibility criteria (MEC)

Using SEC, only 13.1% of patients were eligible for at least one of cabozantinib, regorafenib, or ramucirumab (Figure 1). Expanding eligibility to include patients who met MEC increased the proportion of patients eligible for second‐line treatments to 31.7% (Figure 1). The most common reasons for not meeting SEC across all 3 trials were ECOG ≥2 (61.7%; ECOG 2 29.8%, ECOG 3‐4 31.9%) and Child‐Pugh (CP) ≥B (63.9%; CP‐B7 14.1%, CP‐B8/9 49.8%). Higher ineligibility rates for regorafenib or ramucirumab were likely driven by trial‐specific inclusion criteria, with 28.0% of patients ineligible for regorafenib due to sorafenib intolerance and 58.9% ineligible for ramucirumab due to an AFP < 400.

### 3.4 Survival outcomes

**FIGURE 2:**
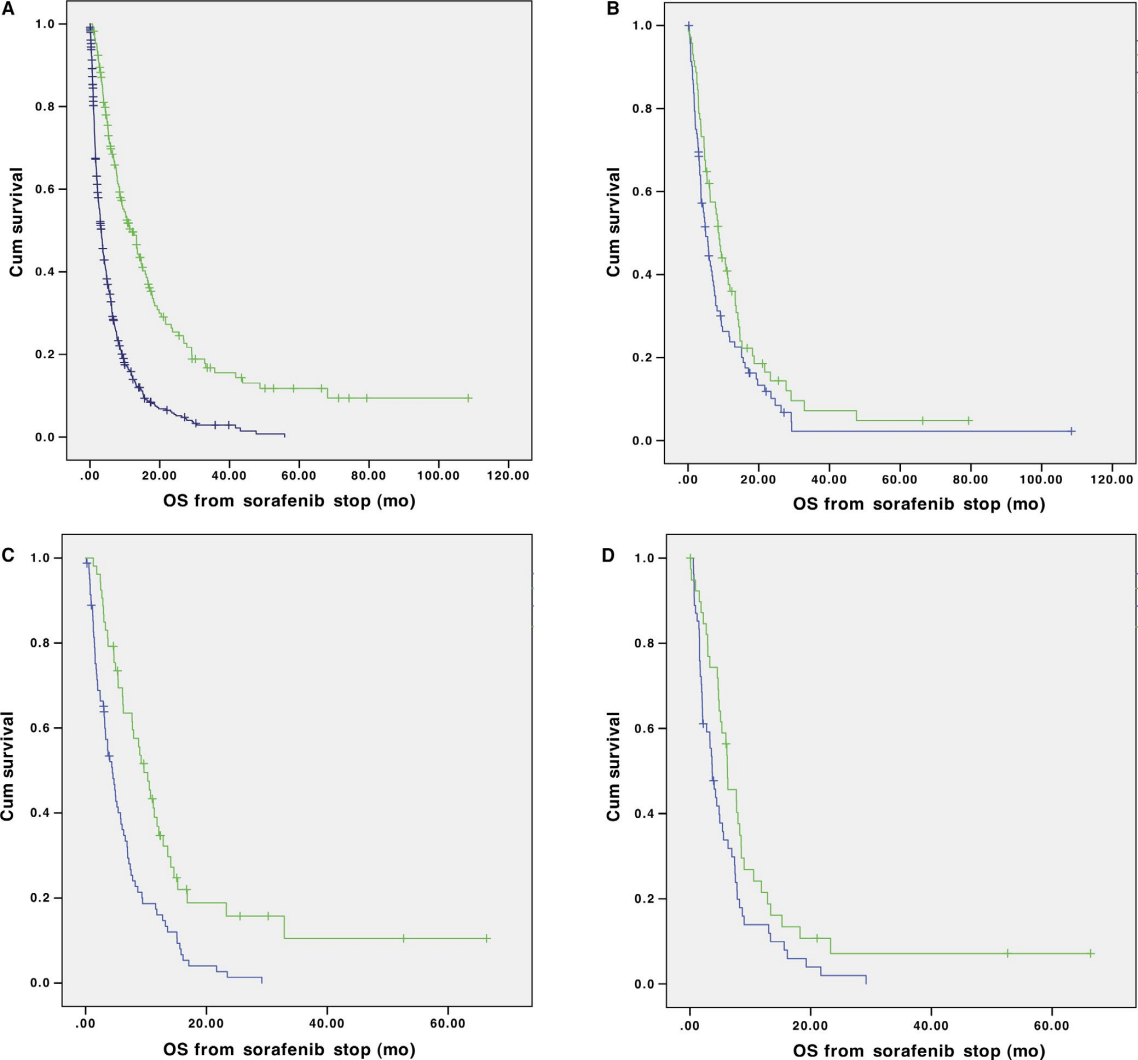
A, Median overall survival was longer (12.1 vs 3.3 mo, *P* < .001) in patients who received subsequent treatment (green) compared to no treatment (blue). Overall survival based on strict eligibility criteria (SEC, green) and modified eligibility criteria (MEC, blue) for B, cabozantinib (8.8 vs 6.2 mo, *P* = .048), C, regorafenib (9.7 vs 6.0 mo, *P* < .001), and D, ramucirumab (6.2 vs 4.9 mo, *P* = .025)

Median OS for HCC patients who were treated with second‐line systemic, localized, or palliative radiation treatment was 10.5, 16.8, and 8.6 months, respectively (*P *< .001). Patients who met SEC were more likely to receive subsequent treatment than those who did not (30.2% vs 9.4%; *P *< .001). Patients who received subsequent treatment had longer median OS than those who did not (12.1 vs 3.3 months; *P *< .001; Figure 2A).

Patients who met SEC for any of the three clinical trials had longer mOS compared to those who were ineligible (8.5 vs 4.0 months, *P *= .001). Median overall survival was also longer if patients met SEC compared to MEC for the CELESTIAL cabozantinib trial (8.8 vs 6.2 months, *P *= .048), RESORCE regorafenib trial (9.7 vs 6.0 months, *P *< .001), and the REACH‐2 ramucirumab trial (6.2 vs 4.9 months, *P *= .025) (Figure 2B‐D). Patients who met MEC for any trial had better mOS if they received subsequent treatment when compared to patients who did not receive treatment (6.0 vs 4.2 months).

### 3.5 Eligibility criteria and subsequent treatment

In a Cox regression model (Table 3), patients with a performance status of ECOG 2 or CP‐B7 have a poorer prognosis than those who met SEC (ECOG HR 1.68, 95% CI 1.37‐2.08, *P *< .001, and CP HR 1.38, 95% CI 1.09‐1.75, *P *= .007). Despite controlling for SEC and MEC, there was continued benefit from systemic (HR 0.45, 95% CI 0.34‐0.61, *P *< .001), localized (HR 0.46, 95% CI 0.32‐0.67, *P *< .001), and palliative (HR 0.41, 95% CI 0.26‐0.63, *P *< .001) treatment.

**TABLE 3:** Cox regression model for overall survival

Category	HR for death	95% CI	*P*‐value
BCLC B or C	0.97	0.81‐1.18	.79
Confirmed Histology	0.58	0.38‐0.86	.007
Sorafenib Intolerance	1.27	0.97‐1.66	.084
Sorafenib Progression	1.62	1.31‐2.01	<.001
AFP > 400	1.66	1.37‐2.01	<.001
ECOG			<.001
0‐1	(ref)		
2	1.68	1.37‐2.08	<.001
3+	2.29	1.77‐2.97	<.001
Subsequent Treatment			<.001
None	(ref)		
Systemic	0.45	0.34‐0.61	<.001
Localized	0.46	0.32‐0.67	<.001
Palliative	0.41	0.26‐0.63	<.001
Child‐Pugh			<.001
A	(ref)		
B7	1.38	1.09‐1.75	.007
B8+	1.8	1.45‐2.24	<.001

The trial‐specific inclusion criteria of sorafenib tolerability in the RESORCE trial selected for potentially better prognostic patients (sorafenib intolerance HR 1.27, 95% CI 0.97‐1.66, *P *< .084), whereas inclusion of only patients who discontinued sorafenib for progression would have selected for a poorer prognosis group (HR 1.62, 95% CI 1.31‐2.01, *P *< .001). The REACH‐2 trial‐specific inclusion criteria of AFP ≥ 400 selected for patients with a poorer prognosis (HR 1.66, 95% CI 1.37‐2.01, *P *< .001).

## 4 DISCUSSION

Over the past decade the lack of subsequent treatment options after progression on first‐line sorafenib likely contributed to the poor outcomes of HCC patients. Our study evaluated subsequent treatments received by HCC patients after sorafenib between 2008 and 2017 and found that a majority of patients (76%) did not receive subsequent treatment. Of those who received treatment after sorafenib, only 13% received systemic therapy and 10% were included in a clinical trial. It is interesting to note that only 13.1% of patients in our study met SEC and would have been eligible for the CELESTIAL, RESORCE, and REACH‐2 trials. Broadening eligibility using MEC, which many physicians would likely use to guide second‐line treatment eligibility in clinical practice, an additional 18.6% of patients could receive subsequent treatment. In addition, this study showed that subsequent treatments appeared to improve survival for patients who met SEC or MEC. In other words, carefully selected patients with performance status ECOG 2 and CP‐B7 liver function may benefit from subsequent treatments.

To our knowledge, this is the first study of HCC patients treated in non‐East Asian countries to characterize subsequent treatments after sorafenib. In addition, it is the only study to examine potential eligibility for novel second‐line treatments postsorafenib. Kondo et al previously reported on 71 HCC patients treated at a Japanese medical center who progressed on sorafenib.^15^ Similar to our findings, Kondo et al showed longer OS and survival postprogression in patients treated with subsequent second‐line or additional treatments (eg, TACE, hepatic arterial infusion chemotherapy (HAIC), combination of tegafur, gimeracil, and oteracil potassium, or clinical trials) after sorafenib. Interestingly, they found that 28 patients (39.4%) received no additional treatment (ie, best supportive care alone) after sorafenib,^15^ which is substantially lower than our study, where 76% of patients received no subsequent treatment.

We found that only a small proportion of HCC patients progressing on sorafenib would be eligible for second‐line regorafenib, cabozantinib, or ramucirumab if strict eligibility criteria (SEC) from their respective clinical trials were followed. In clinical practice, it is likely safe to treat patients with a performance status of ECOG 2 and/or Child‐Pugh‐B liver function with an oral multikinase inhibitor. The SHARP trial comparing first‐line sorafenib to placebo included 8% of patients with a performance status of ECOG 2 and 5% of patients with Child‐Pugh‐B liver function in the sorafenib arm.^5^ In an exploratory subgroup analysis of the SHARP trial, patients had a better overall survival with sorafenib whether they had a performance status of ECOG 0, 1, or 2.^16^ Similarly, in a subgroup analysis of the Asia Pacific trial, treatment with sorafenib was associated with better overall survival compared to placebo irrespective of ECOG performance status (ECOG 0, 1 or 2) or liver function (normal or elevated ALT/AST, bilirubin, or AFP).^17^ GIDEON was a noninterventional surveillance study which aimed to evaluate safety of sorafenib in real‐world HCC patients, particularly patients who were not well represented in randomized clinical trials.^18^ Approximately 28% of patients on this study had Child‐Pugh‐B liver function. There were more Child‐Pugh‐A patients treated for >8 weeks than Child‐Pugh‐B patients (65% vs 42%) and there was a higher rate of treatment discontinuation due to adverse effects in Child‐Pugh‐B patients compared to Child‐Pugh‐A patients (40% vs 25%); however, the safety profile was similar between Child‐Pugh‐A and B patients.^18^ Unfortunately, none of the positive second‐line clinical trials included patients with a performance status of ECOG 2 or Child‐Pugh‐B liver function,^12^, ^13^, ^14^ even though real‐world data suggest that these patients may be well enough for treatment. Data regarding the safety and efficacy of regorafenib, cabozantinib, and ramucirumab in these patient populations are unlikely to be examined in future clinical trials and will most likely be generated in studies of real‐world treatment outcomes. The current study suggests that treatment of patients with ECOG 2 performance status or Child‐Pugh‐B7 may result in better survival outcomes compared to no further treatment.

Our study also confirms the relative prognostic impact of the trial‐specific eligibility criteria used in RESORCE and REACH‐2. Patients were required to have evidence of radiographic progression on sorafenib, as well as demonstrated sorafenib tolerability to be eligible for the RESORCE trial. On multivariable analysis, we found that radiographic progression on sorafenib was associated with worse prognosis (HR 1.622, 95% CI 1.312‐2.006, *P *< .001). On the other hand, we found potentially better prognosis for patients who demonstrated sorafenib tolerability (HR 1.268 for sorafenib intolerance, 95% CI 0.969‐1.660, *P *< .084). The REACH‐2 trial excluded patients with an AFP < 400. We showed in multivariable analysis that patients with an AFP ≥ 400 had worse prognosis (HR 1.663, 95% CI 1.375‐2.011, *P *< .001), which likely contributed to the shorter survival in patients who met eligibility for REACH‐2.

The first‐line treatment of HCC is also rapidly evolving. Recently, the REFLECT trial showed that another multikinase inhibitor, lenvatinib, is noninferior to sorafenib in the first‐line systemic treatment of HCC for overall survival.^7^ In addition, the IMbrave 150 trial recently showed a survival benefit from treatment with atezolizumab plus bevacizumab when compared to sorafenib in the first‐line setting.^11^ Lenvatinib is now a first‐line option in the treatment of HCC and atezolizumab plus bevacizumab will soon be an option as well. Studies evaluating real‐world eligibility for these new first‐line treatments and outcomes with subsequent second‐line treatments are planned and will likely be of great interest to physicians involved in HCC treatment.

This study has several limitations. First, it is a retrospective analysis as opposed to a prospective study. However, this was a relatively large retrospective study of consecutive patients from multiple cancer centers across Canada, which mitigates potential selection bias. Second, survival outcomes in patients who received subsequent treatment could have been impacted by patient selection bias; however, we tried to adjust for this in the multivariable analysis. Finally, since the patient population studied was during a time when the benefit of second‐line treatments was unclear, the patient cohort included only a limited number of patients who received cabozantinib or regorafenib on clinical trials and no patients received ramucirumab. Due to these small numbers, we were unable to complete a robust analysis of outcomes for patients who received one of these agents. Further analyses evaluating outcomes of patients receiving subsequent treatment after sorafenib based on SEC vs MEC would be useful, and should be further elucidated as treatments such as cabozantinib, regorafenib, or ramucirumab become more routinely used in clinical practice. However, this population can serve as the comparator for future studies examining these new second‐line systemic treatments for HCC.

## 5 CONCLUSION

In summary, over the past decade very few HCC patients received second‐line systemic treatment after sorafenib due to lack of effective options. We found that the majority of real‐world patients would not have been eligible for regorafenib, cabozantinib, or ramucirumab if strict eligibility criteria as defined in their respective trials were used to guide clinical treatment. Our study also suggests that certain patients who would have been ineligible based on an ECOG 2 performance status or Child‐Pugh‐B7 liver function may still potentially benefit from subsequent treatment. Future clinical trials should consider utilizing these broadened, modified eligibility criteria so that their patient population better represents those who are treated in routine clinical practice. Ongoing real‐world evidence generation will be important to evaluate outcomes in these understudied patient groups.

## CONFLICT OF INTEREST

Lee‐Ying—Consulting: BMS, Eisai, Merck, Bayer, Janssen, Sanofi. Research funding (To institution) Sanofi, Clinical Trials (To Institution) Boston Biomedical. Tam—Honoraria: BMS, Eisai, Ipsen. Research Funding (To Institution): Bayer, Eisai. Clinical Trials Funding (To Institution): AstraZeneca, BMS, Boston Biomedical, Exelixis, Merck. Davies—Clinical trial funding from Merck, BMS. Honoraria/Consulting: Eisai. Knox—Research support: Merck, Roche, AstraZeneca, Ipsen. Consulting: Merck.

## AUTHOR CONTRIBUTIONS

Andrea S. Fung: Formal analysis, writing original draft, reviewing and editing of manuscript. Vincent C. Tam: conceptualization, data curation, methodology, funding acquisition, supervision, reviewing, and editing. Daniel E. Meyers: Data curation, methodology, reviewing, and editing. Hao‐Wen Sim, Jennifer J. Knox, Valeriya Zaborska, Janine Davies, Yoo‐Joung Ko, Eugene Batuyong, Haider Samawi, and Winson Y. Cheung: Data curation, reviewing, and editing. Richard Lee‐Ying: conceptualization, data curation, formal analysis, methodology, funding acquisition, supervision, reviewing, and editing.
